# Supplementary material for: Psychometric Performance of a Condition-Specific Quality-of-Life Instrument for Dutch Children Born with Esophageal Atresia
Source: Children (Basel). 2022 Oct 1;9(10):1508. doi: 10.3390/children9101508 (PMC9600375; doi:10.3390/children9101508)
Supplement: Supplementary file 1 [file children-09-01508-s001.zip › children-1856299-supplementary.pdf]

Additional files of the manuscript:

Psychometric performance of a condition-specific quality of life instrument for Dutch children born with esophageal atresia

Chantal A. ten Kate[a], Hanneke IJsselstijn[a], Michaela Dellenmark-Blom[b], E. Sofie van Tuyll van Serooskerken[c], Maja Joosten[d], René M.H. Wijnen[a], Michiel P. van Wijk[e], on behalf of the DCEA Study Group

[a] Department of Pediatric Surgery and Intensive Care Children, Erasmus University Medical Centre - Sophia Children's Hospital, Rotterdam, the Netherlands
[b] Department of Pediatric Surgery, Queen Silvia Children's Hospital, Sahlgrenska University Hospital, Göteborg, Sweden
[c] Department of Pediatric Surgery, Wilhelmina Children's Hospital, University Medical Center Utrecht, Utrecht, the Netherlands
[d] Department of Pediatric Surgery, Radboud University Medical Center, Amalia Children's Hospital, Nijmegen, the Netherlands
[e] Department of Pediatric Gastroenterology and Nutrition, Amsterdam UMC – Emma Children's Hospital, University of Amsterdam, Amsterdam, the Netherlands

Corresponding author:
Michiel P. van Wijk, MD PhD, m.vanwijk@amsterdamumc.nl

Content:

File S1. Description of measurement instruments
File S2. Detailed methodological description of the translation, cognitive debriefing and field testing phases
File S3. Cognitive debriefing
File S4. Item evaluation of the field test
File S5. Reliability of the field test
File S6. Results of the PedsQL questionnaire

DCEA Study Group – pediatric surgeons and pediatric gastroenterologists of the participating centers:

- Erasmus University Medical Centre - Sophia Children's Hospital, Rotterdam, The Netherlands: Erasmus University Medical Centre - Sophia Children's Hospital: R.M.H. (René) Wijnen, J. (John) Vlot, J.M. (Marco) Schnater, H. (Hanneke) IJsselstijn, B.A.E. (Barbara) de Koning.
- Wilhelmina Children's Hospital, University Medical Center Utrecht, Utrecht, The Netherlands: D.C. (David) van der Zee, S.H.A.J. (Stefaan) Tytgat, M.Y.A. (Maud) Lindeboom, R.H.J. (Roderick) Houwen, A. (Annemone) van den Berg
- Radboud University Medical Center, Amalia Children's Hospital, Nijmegen, The Netherlands: S.M.B.I. (Sanne) Botden, H. (Horst) Scharbatke, M. (Maarten) Schurink, G. (Gerard) Damen, N. (Nicole) Gierenz
- Amsterdam UMC – Emma Children’s Hospital, University of Amsterdam, Amsterdam, The Netherlands: E. (Ernst) van Heurn, M.W. (Matthijs) Oomen, S. (Sander) Zwaveling, S. (Sjoerd) de Beer, R. (Ramon) Gorter, M.P. (Michiel) van Wijk

File S1. Description of measurement instruments

The EA-QOL© questionnaires[1]
The EA-QOL© questionnaire for children aged 2-7 years old (proxy-report) consists of 17 items in three domains: eating (7 items), physical health and treatment (6 items), and social isolation and stress (4 items). The EA-QOL© questionnaire for children aged 8-17 years old (proxy-report and self-report) consists of 24 items in four domains: eating (8 items), social relationships (7 items), body perception (5 items) health and well-being (4 items). The total score is calculated from all items (respectively 17 or 24) together. Subscales and total scores can only be calculated if ≤30% of the items is missing. All items are asking about problems in the past 4 weeks, and are answered on a 5-point Likert scale. Total and subscale scores are rescaled to a score between 0 (worst) and 100 (best).

The Paediatric Quality of Life Inventory™ 4.0 (PedsQL) questionnaire[2]
The PedsQL questionnaire is available in different age-appropriate versions. For children aged 2-4 years old, the parent-proxy-report version consists of 21 questions within four domains: physical functioning (8 items), emotional functioning (5 items), social functioning (5 items), and school functioning (3 items). A fifth domain, psychosocial health, is calculated as the sum of the emotional, social, and school functioning subscales.

For children aged 5-7 years old, a parent-proxy-report and self-report version is available. In this study, we only used the parent-proxy-report, which consists of 23 questions within four domains: physical functioning (8 items), emotional functioning (5 items), social functioning (5 items), and school functioning (5 items). A fifth domain, psychosocial health, is calculated as the sum of the emotional, social, and school functioning subscales.

For children aged 8-12 years old and 13-17 years old, age-specific proxy-reports and self-reports are available. We used both the proxy-report and the self-report for these age groups in this study. Both versions consist of 23 questions within four domains: physical functioning (8 items), emotional functioning (5 items), social functioning (5 items), and school functioning (5 items). A fifth domain, psychosocial health, is calculated as the sum of the emotional, social, and school functioning subscales.

The total score is calculated from all items together. Subscales and total scores can only be calculated if ≤50% of the items is missing. All items are asking about problems in the past 4 weeks, and are answered on a 5-point Likert scale. Total and subscale scores are rescaled to a score between 0 (worst) and 100 (best).

File S2. Detailed methodological description of the translation, cognitive debriefing and field testing phases

Ethical approval

The study has been approved by the participating institutional review boards of the Erasmus Medical Center (MEC-2019-0521), the University Medical Center Utrecht (MEC-20-564/C), the Radboud University Medical Center (MEC-2020-6961) and the University of Amsterdam (MEC-2019-631).

Translation

A Swedish-Dutch forward-backward translation has been conducted according to the 'Translation and Cultural Adaptation of Patient Reported Outcomes Measures - Principles of Good Practice' [3]. Two forward translations have been developed by two certified native Dutch-speaking translators, independently, who were recruited from a professional agency. To avoid any ambiguities, they were provided with a document containing the explanations and concepts of the questions. After review of the translations by two members of the research team (CtK and MvW), a reconciliation meeting was planned together with one of the translators wherein discrepancies between the two forward translations were solved. All reconciliation decisions were documented.

Next, one backward translation was performed by a third certified translator recruited from the professional agency, who was a native Swedish speaker and fluent in the Dutch language and who had not seen the original questions. To ensure the conceptual equivalence, this backward translation was reviewed by the original, Swedish developer (MDB) of the EA-QOL© questionnaire. During a virtual cross-cultural meeting between CtK, MvW and MDB, all translations were systematically discussed and the last discrepancies were solved.

Cognitive debriefing

To ensure that the instructions, items and response scale were understood by the respondents as intended, cognitive debriefing was applied through face-to-face interviews with children with EA and one of their parents [4]. To avoid exchange of information about the questionnaire, interviews with 8-to-17-year old children were performed with the child separated from the parent, simultaneously at the same time in different rooms.

Three groups were selected: group A) parents of children with EA between the age of 2 and 7 years old (proxy-report), group B1) parents of children with EA between the age of 8 and 17 years old (proxy-report), and group B2) children with EA between the age of 8 and 17 years old (self-report).

To assure the representation of the EA population, each group consisted of a predefined stratified sample of children who were categorized into patients with mild, moderate or severe complaints (Supplementary Table 1) modified from the severity criteria used in the pilot testing in Sweden[5]. Children in the category 'mild' did not suffer from additional associated anomalies and had no complaints at all, or only one of the following symptoms: dysphagia, gastroesophageal reflux disease (GERD), or a chronic pulmonary condition for which medication is not required (for example upper respiratory tract infections that do not require antibiotic treatment). Children with associated anomalies, automatically fell

into the category ‘moderate’ or ‘severe’. Furthermore, children in the category ‘moderate’ had one or more of the following symptoms: actual dysphagia, GERD, a history of esophageal dilatation, or a chronic pulmonary condition for which daily medication was required. Children in the category ‘severe’ had all of the above medical symptoms: actual dysphagia, GERD, a history of esophageal dilatation and clinically significant chronic pulmonary condition.

The interviews were held during an annual meeting of the Dutch patient support group VOKS (Vereniging voor Ouderen en Kinderen met een Slokdarmatresie). Participants were initially approached by a member of the board of the patient support group by telephone in the weeks prior to the meeting. If participants were interested, a member of the research team called one of the parents to explain the aim of the study. After verbal informed consent, the researcher recorded the presence of the above-mentioned medical conditions. Children were categorized into ‘mild’, ‘moderate’ or ‘severe’ based on the parental proxy-report obtained during this initial telephone call.

Participants filled out the questionnaire on paper at the same time as they gave verbal feedback on the clarity and adequacy of the items. They indicated if an item was easy to understand, and if an item was sensitive for answer, for example because it raised certain emotions like sadness or fear. If an item could not be answered because it was not a applicable to a child’s situation, too difficult, or too sensitive, the item was left empty and registered as ‘missing’. Finally, they were asked if they had missed any items in this questionnaire. Field notes were made by the interviewer, including observations of non-verbal language. In 8-to-17-year-olds, the interviews were performed individually, separately and simultaneously with the child and the parent. The child and the parent were not able to exchange information about the questionnaire. If two parents were present, only one parent participated in the cognitive debriefing. Parents were free to decide themselves which parent would participate. The interviewer was either a member of the research team (CtK or MvW) or another researcher in the field of EA. None of the interviewers was involved as care provider of the participants.

The results from the cognitive debriefing were analyzed using manifest content analysis. The participants’ understanding of the items was compared with the predefined explanations and concepts of the items. This content analysis was performed by two members of the research team (CtK and MvW) and discussed during a meeting with the original developer (MDB). Instructions and items were adjusted if necessary until consensus was reached.

Field testing

Finally, the feasibility, validity and reliability of the translated questionnaires was statistically evaluated. Participants were recruited via the routine care of four university hospitals in The Netherlands. Participants were eligible if they had sufficient command of the Dutch language, and the child was aged 2-17 years old and born with EA. Children with known intellectual disability were excluded. They either did not get an invitation, or were excluded afterwards if parents informed us after filling out the questionnaire.

All participants were invited to participate through a personal letter. Children ≥12 years old received their own personal letter, since from this age forward children have to consent to participate in research

themselves as well. Only one parents filled out the questionnaire; parents were free to decide who of them would participate.

The letter contained a personal code, which gave access to the online questionnaires (LimeSurvey GmbH version 2.06lts, Hamburg, Germany). By filling out the questionnaires, participants automatically gave consent for the study. This was also explained in the invitation letter. All parents and children aged ≥8 years old filled out the age-appropriate version of the Dutch-translated EA-QOL© questionnaire and the PedsQL questionnaire [2]. Additionally, all parents and children aged ≥12 years old filled out a short questionnaire on sociodemographic items and presence of digestive symptoms, feeding difficulties and respiratory symptoms in the past 4 weeks. To maximize the response rate, parents and/or patients received maximally two reminders. With the last reminder, they also received the questionnaires on paper with a pre-stamped envelope for reply.

To examine the reliability of the EA-QOL© questionnaires over time, all parents and/or patients who participated in the initial test received a second invitation letter three weeks after the initial response. This letter contained a new personal code, which was used to fill out the EA-QOL© questionnaires a second time. Participants were also asked about potential differences in the presence of digestive symptoms, feeding difficulties and respiratory symptoms during the recall period. Participants again received a maximum of two reminders.

File S3. Cognitive debriefing

	Clinical characteristics of the patient	Group A (2-7 years)		Group B (8-17 years)	
		Predefined	*Included (n=9)*	*Predefined*	*Included (n=10)*
**Mild**	No associated anomalies and no complaints at all<br>*or*<br>No associated anomalies and one of the following symptoms:<br>▪ Dysphagia<br>▪ GERD<br>▪ Chronic pulmonary condition for which medication is not required	2 patients	2 patients	2 patients	4 patients
**Moderate**	Associated anomalies<br>*or*<br>One or more of the following symptoms:<br>▪ Dysphagia<br>▪ GERD<br>▪ History of esophageal dilatation<br>▪ Chronic pulmonary condition for which daily medication is required	4-5 patients	5 patients	4-5 patients	4 patients
**Severe**	Associated anomalies<br>*and*<br><u>All</u> of the following symptoms:<br>▪ Dysphagia<br>▪ GERD<br>▪ History of esophageal dilatation<br>▪ Chronic pulmonary condition for which daily medication is required	2-3 patients	2 patients	2-3 patients	2 patients

Supplementary Table S1. Stratified sample size for the cognitive debriefing. Children were categorized into ‘mild’, ‘moderate’ or ‘severe’ based on parental proxy-report obtained during a telephone call with the researcher prior to the cognitive debriefing interviews. Predefined = ideal number of participating patients based on the study protocol and normal distribution in the esophageal atresia population, included = actual number of participating patients. Group A = children with esophageal atresia aged 2-7 years old, group B = children with esophageal atresia aged 8-17 years old. GERD = gastroesophageal reflux disease

		Aim of the item, to measure perceived impact on… (reference)	Response (n)						Easy to understand (n) [A]	Sensitive to answer (n) [B]	Important comments
			Never	*Seldom*	*Sometimes*	*Often*	*Always*	*Missing* [C]			
**2-7 years old (parent-report, n=9)**	**Q1**	Eating problems, due to food sticking in the throat (Q1)	1	2	4	1	0	1	7 (78%)	2 (22%)	My child is not fed orally, only tube feeding.
	**Q2**	The child's ability to eat a large portion or full meal (Q2)	0	1	4	2	1	1	7 (78%)	1 (11%)	
	**Q3**	Eating-related stress on the child (Q3)	4	1	3	1	0	0	9 (100%)	0 (0%)	
	**Q4**	The child's satisfaction with their eating pace (Q4)	2	2	4	0	1	0	7 (78%)	0 (0%)	Differs between home and school. Time pressure experienced by the parents or the child itself?
	**Q5**	Emotional impact, in terms of worry, of the risk of choking on food (Q5)	6	1	1	0	1	0	8 (89%)	0 (0%)	
	**Q6**	Degree of the child's problem with vomiting (Q7)	5	1	1	0	2	0	8 (89%)	0 (0%)	My child never vomits.
	**Q7**	Limitations on social activities and events that include eating with peers (Q8)	2	1	1	4	0	1	7 (78%)	1 (11%)	
	**Q8**	Physical limitation on playing games or sports (Q9)	3	1	3	2	0	0	9 (100%)	1 (11%)	
	**Q9**	Physical ability of the child to perform physically demanding activities in daily life compared to healthy peers (Q10)	3	0	3	2	1	0	9 (100%)	1 (11%)	
	**Q10**	Respiratory symptoms on the child's daily life (Q11)	1	2	5	1	0	0	9 (100%)	1 (11%)	
	**Q11**	Problematic respiratory infections on the child's daily life (Q12)	2	3	3	1	0	0	6 (67%)	1 (11%)	
	**Q12**	Severity of the emotional impact of the need for medical treatment (Q13)	6	1	1	0	1	0	9 (100%)	0 (0%)	All medication is administered by gastrostomy tube.
	**Q13**	EA related morbidity on the child's sleep (Q14)	3	2	3	1	0	0	8 (89%)	2 (22%)	
	**Q14**	School absence and social isolation with those of peers (Q15)	3	2	4	0	0	0	7 (78%)	1 (11%)	
	**Q15**	Social stress of explaining one's condition to other people at very young age (Q16)	6	0	1	0	1	1	8 (89%)	0 (0%)	My child is too young to explain this.
	**Q16**	Social stigma and stress in the child (Q17)	3	1	4	0	0	1	8 (89%)	1 (11%)	
	**Q17**	Social exclusion and stress in the child's life (Q18)	6	0	1	0	0	2	6 (67%)	0 (0%)	My child is too young to understand this.

**8-17 years old (parent-report, n=10)**	**Q1**	Food getting stuck in the throat, from the child's perspective (Q1)	3	4	1	0	0	2	10 (100%)	2 (20%)	My child is not fed orally, only parenterally.
	**Q2**	Restriction on food intake on the child (Q2)	2	3	1	0	2	2	7 (70%)	3 (30%)	
	**Q3**	Pain during food and fluid intake due to the child's condition (Q3)	5	0	1	1	0	3	9 (90%)	1 (10%)	
	**Q4**	The need to drink a lot when eating (Q4)	3	0	2	0	2	3	5 (50%)	3 (30%)	Does my child need to drink to be able to eat or do I need to remind my child?
	**Q5**	Emotional impact of fear of choking (Q5)	5	3	0	0	0	2	9 (90%)	1 (10%)	My child never chokes.
	**Q6**	Impact of choking on the child's eating situation (Q6)	3	3	0	0	0	4	8 (80%)	2 (20%)	
	**Q7**	Child's ability to eat at the same pace as children their own age, from their perspective (Q7)	0	1	0	0	6	3	7 (70%)	1 (10%)	
	**Q8**	The degree of the child's problem with vomiting (Q8)	1	1	0	0	2	6	6 (60%)	2 (20%)	My child is unable to vomit due to fundoplication surgery.
	**Q9**	Experience of emotional isolation (Q9)	3	3	1	1	0	2	8 (80%)	0 (0%)	
	**Q10**	Social stress related to severity of explaining EA to others (Q10)	2	2	4	0	0	2	9 (90%)	1 (10%)	
	**Q11**	Social exclusion in terms of being called names by others (Q11)	4	3	1	0	0	2	10 (100%)	1 (10%)	
	**Q12**	Social exclusion in terms of perceiving that others stare at you (Q12)	3	2	2	1	0	2	10 (100%)	1 (10%)	
	**Q13**	Social stress related to the need to explain their scar(s) to other people (Q13)	4	3	1	0	0	2	10 (100%)	0 (0%)	My child never gets any questions.
	**Q14**	Children's perception of others saying mean things about them from the perspective of social exclusion (Q14)	5	2	1	0	0	2	9 (90%)	3 (30%)	
	**Q15**	Strain of reacting to other people's questions (Q16)	4	3	1	0	0	2	10 (100%)	0 (0%)	
	**Q16**	Experience of feeling different due to surgical scar(s) (Q17)	3	2	2	1	0	2	10 (100%)	0 (0%)	
	**Q17**	Concern regarding what to wear because of the surgical scar(s) (Q18)	4	0	3	1	0	2	10 (100%)	0 (0%)	
	**Q18**	Unease related to the scar(s) being visible to others (Q19)	4	0	2	2	0	2	10 (100%)	2 (20%)	
	**Q19**	Impact of scar(s) on children's perception of their looks (Q20)	3	3	1	1	0	2	9 (90%)	2 (20%)	
	**Q20**	Impact of being small for age (Q21)	1	1	2	0	0	6	9 (90%)	3 (30%)	My child is not smaller than other children of his/her age.
	**Q21**	Impact of breathing difficulties on exercise and play, from the perspective of physical performance (Q23)	3	0	2	3	0	2	10 (100%)	1 (10%)	
	**Q22**	Impact of EA related morbidity on sleep (Q24)	3	3	2	0	0	2	10 (100%)	0 (0%)	
	**Q23**	Emotional impact with regard to	5	2	1	0	0	2	10 (100%)	1 (10%)	

		worries about the future due to EA (Q25)									
	Q24	Emotional impact of EA in terms of feelings of sadness (Q26)	5	2	0	0	1	2	9 (90%)	1 (10%)	
8-17 years old (self-report, n=10)	Q1	Food getting stuck in the throat, from the child's perspective (Q1)	2	3	3	0	0	2	6 (60%)	1 (10%)	Food never gets stuck.
	Q2	Restriction on food intake on the child (Q2)	5	1	1	1	1	1	10 (100%)	1 (10%)	
	Q3	Pain during food and fluid intake due to the child's condition (Q3)	6	1	2	0	0	1	10 (100%)	2 (20%)	
	Q4	The need to drink a lot when eating (Q4)	4	1	1	1	1	1	8 (80%)	2 (20%)	
	Q5	Emotional impact of fear of choking (Q5)	4	3	3	0	0	0	8 (80%)	0 (0%)	
	Q6	Impact of choking on the child's eating situation (Q6)	8	1	1	0	0	1	8 (80%)	0 (0%)	
	Q7	Child's ability to eat at the same pace as children their own age, from their perspective (Q7)	2	1	2	1	3	1	10 (100%)	1 (10%)	
	Q8	The degree of the child's problem with vomiting (Q8)	4	2	1	1	1	1	5 (50%)	2 (20%)	I never have to vomit.
	Q9	Experience of emotional isolation (Q9)	6	3	1	0	0	0	10 (100%)	0 (0%)	
	Q10	Social stress related to severity of explaining EA to others (Q10)	1	3	4	1	1	0	9 (90%)	1 (10%)	
	Q11	Social exclusion in terms of being called names by others (Q11)	8	2	0	0	0	0	10 (100%)	2 (20%)	
	Q12	Social exclusion in terms of perceiving that others stare at you (Q12)	5	2	2	1	0	0	10 (100%)	0 (0%)	
	Q13	Social stress related to the need to explain their scar(s) to other people (Q13)	8	1	1	0	0	0	9 (90%)	1 (10%)	Nobody can see my scars, they are covered by my swimming suit.
	Q14	Children's perception of others saying mean things about them from the perspective of social exclusion (Q14)	8	2	0	0	0	0	9 (90%)	1 (10%)	
	Q15	Strain of reacting to other people's questions (Q16)	4	1	4	0	0	1	9 (90%)	1 (10%)	
	Q16	Experience of feeling different due to surgical scar(s) (Q17)	9	0	1	0	0	0	10 (100%)	0 (0%)	
	Q17	Concern regarding what to wear because of the surgical scar(s) (Q18)	7	1	0	1	1	0	8 (80%)	1 (10%)	
	Q18	Unease related to the scar(s) being visible to others (Q19)	7	0	3	0	0	0	9 (90%)	0 (0%)	
	Q19	Impact of scar(s) on children's perception of their looks (Q20)	9	1	0	0	0	0	9 (90%)	1 (10%)	
	Q21	Impact of breathing difficulties on exercise and play, from the perspective of physical performance (Q23)	2	2	0	4	1	1	9 (90%)	0 (0%)	
	Q22	Impact of EA related morbidity on sleep (Q24)	7	1	1	0	1	0	8 (80%)	1 (10%)	
	Q23	Emotional impact with regard to	8	1	0	0	0	1	9 (90%)	0 (0%)	I never think about the future.

		worries about the future due to EA (Q25)									
	**Q24**	Emotional impact of EA in terms of feelings of sadness (Q26)	7	2	1	0	0	0	9 (90%)	1 (10%)	

Supplementary Table S2. Summary of the results of the cognitive debriefing. The bold item numbers correspond with the items of the Dutch EA-QOL questionnaire. The item numbers in brackets after the aim of the item correspond with the items of the original Swedish-German pilot questionnaire before item reduction. The complete English questions can be found in the supplementary material of the original article[5]. The last column shows a selection of the most important comments on the items, which have led to a change. Q20 is missing from the self-reports for 8-17 years old, due to administrative problems. [A] Answered with yes or no. [B] For example because it raised certain emotions like sadness or fear, answered with yes or no. [C] If an item could not be answered because it was not a applicable to a child’s situation, too difficult, or too sensitive, the item was left empty and registered as ‘missing’.

Group A	Group B1	Group B2
Physical height<br>Hospital visits, hospitalizations<br>Trauma and need for EMDR therapy<br>(Self-imposed) dietary restrictions	Physical condition, exercise endurance, lung capacity<br>Comorbidities, associated anomalies<br>Medical equipment (gastrostomy, aerosol therapy, central venous line)<br>Hospital visits, hospitalizations<br>Concerns about a child's psychosocial wellbeing (trauma)<br>Social contacts<br>Cultural differences, challenges for immigrants<br>Transition to adulthood	Visible deformities<br>Comorbidities, associated anomalies<br>Coughing in public, using medication in public

Supplementary Table S3. Overview of topics parents and children missed in the EA-QOL© questionnaire. Group A = parents of children with EA aged 2-7 years old (proxy-report) Group B1 = parents of children with EA aged 8-17 years old (proxy-report), group B2 = children with EA aged 8-17 years old (self-report).EMDR = eye movement desensitization and reprocessing

File S4. Item evaluation of the field test

		Topic (reference)	Missing values, n (%)	Mean ± SD	Range	Skewness	Kurtosis
2-7 years old proxy-report (n=101)	**Q1**	Food getting stuck (Q1)	-	2.2 ± 0.9	1-5	0.6	0.2
	**Q2**	Eating full meals (Q2)	1 (1.0)	2.3 ± 1.3	1-5	0.7	-0.6
	**Q3**	Eating is stressful (Q3)	-	1.5 ± 0.9	1-5	2.0	4.3
	**Q4**	Pace of eating (Q4)	5 (5.0)	4.0 ± 1.1	1-5	-1.0	0.5
	**Q5**	Choking (Q5)	7 (6.9)	1.6 ± 0.9	1-5	1.7	2.6
	**Q6**	Vomiting (Q7)	9 (8.9)	1.9 ± 1.0	1-5	1.0	0.6
	**Q7**	Eating with friends (Q8)	5 (5.0)	2.0 ± 1.2	1-5	1.1	0.2
	**Q8**	Tired (Q9)	1 (1.0)	2.2 ± 1.2	1-5	0.6	-0.7
	**Q9**	Strength (Q10)	5 (5.0)	2.2 ± 1.2	1-5	0.8	-0.5
	**Q10**	Respiratory problems (Q11)	-	2.2 ± 1.1	1-5	0.4	-1.0
	**Q11**	Respiratory infections (Q12)	22 (21.8)	2.0 ± 1.1	1-5	0.7	-0.7
	**Q12**	Medicine (Q13)	16 (15.8)	1.9 ± 1.0	1-5	1.0	0.8
	**Q13**	Sleeping (Q14)	1 (1.0)	1.6 ± 0.9	1-5	1.7	2.5
	**Q14**	Absence from school (Q15)	23 (22.8)	1.7 ± 1.0	1-5	1.4	1.5
	**Q15**	Explaining to others (Q16)	33 (32.7)	1.7 ± 1.0	1-5	1.4	1.4
	**Q16**	Comments (Q17)	26 (25.7)	1.6 ± 1.0	1-4	1.2	-0.0
	**Q17**	Noises (Q18)	25 (24.8)	1.6 ± 0.9	1-4	1.2	0.4

Supplementary Table S4. Feasibility of the EA-QOL© questionnaire for 2-7 year old children (proxy-report, n=101). The bold item numbers correspond with the items of the Dutch EA-QOL questionnaire. The item numbers number in brackets after the topics correspond with the items of the original Swedish-German pilot questionnaire before item reduction. The complete English questions can be found in the supplementary material of the original article[5]. Items were answered on a 5-point Likert scale, ranging from never (1) to always (5). Raw, untransformed scores are presented in this table. Feasibility (percentage of items with >5% missing values[5]) was considered poor (>30%), moderate (10-30%) or good (<10%).

		Topic (reference)	Missing values, n (%)	Mean ± SD	Range	Skewness	Kurtosis
8-17 years old proxy-report (n=139)	**Q1**	Food getting stuck (Q1)	27 (19.9)	2.8 ± 1.4	1-5	0.4	-1.0
	**Q2**	Restricting from food (Q2)	3 (2.2)	1.6 ± 0.9	1-5	1.6	2.6
	**Q3**	Pain (Q3)	6 (4.4)	1.6 ± 0.8	1-5	1.4	1.8
	**Q4**	Drinking (Q4)	2 (1.5)	2.4 ± 1.4	1-5	0.6	-1.0
	**Q5**	Afraid of choking (Q5)	3 (2.2)	1.3 ± 0.7	1-5	2.7	8.6
	**Q6**	Hard to eat due to choking (Q6)	5 (3.7)	1.2 ± 0.5	1-3	2.3	4.7
	**Q7**	Pace of eating (Q7)	1 (0.7)	3.6 ± 1.4	1-5	-0.6	1.0
	**Q8**	Vomiting (Q8)	39 (28.7)	1.4 ± 1.0	1-5	2.5	6.0
	**Q9**	Loneliness (Q9)	8 (5.9)	1.8 ± 1.2	1-5	1.2	0.3
	**Q10**	Explaining to others (Q10)	3 (2.2)	1.8 ± 1.1	1-5	1.2	0.6
	**Q11**	Name-calling (Q11)	1 (0.7)	1.2 ± 5.2	1-4	3.3	12.5
	**Q12**	Staring (Q12)	3 (2.2)	1.7 ± 1.0	1-5	1.1	0.0
	**Q13**	Scars (Q13)	8 (5.8)	1.4 ± 0.8	1-4	1.9	2.7
	**Q14**	Saying mean things (Q14)	1 (0.7)	1.2 ± 0.5	1-3	2.2	3.7
	**Q15**	Feeling awkward (Q16)	3 (2.2)	1.4 ± 0.8	1-5	2.0	3.6
	**Q16**	Feeling different (Q17)	1 (0.7	1.5 ± 0.9	1-5	1.8	2.1
	**Q17**	Adjusting cloths (Q18)	2 (1.5)	1.4 ± 0.9	1-5	1.8	3.8
	**Q18**	Visible scars (Q19)	3 (2.2)	1.4 ± 0.8	1-5	1.9	2.7
	**Q19**	Feeling imperfect (Q20)	3 (2.2)	1.4 ± 0.8	1-5	2.5	6.4
	**Q20**	Smaller than peers (Q21)	28 (20.6)	1.6 ± 1.0	1-5	1.5	1.2
	**Q21**	Breathing difficulties (Q23)	1 (0.7)	2.0 ± 1.1	1-5	0.9	-0.1
	**Q22**	Sleeping (Q24)	4 (2.9)	1.7 ± 1.0	1-5	1.2	0.8
	**Q23**	Worried about future (Q25)	4 (2.9)	1.2 ± 0.6	1-5	3.4	13.8
	**Q24**	Sad (Q26	2 (1.5)	1.3 ± 0.6	1-3	2.1	3.1
8-17 years old self-report (n=131)	**Q1**	Food getting stuck (Q1)	15 (11.5)	3.0 ± 1.5	1-5	0.1	-1.3
	**Q2**	Restricting from food (Q2)	-	1.6 ± 0.9	1-5	1.6	1.7
	**Q3**	Pain (Q3)	2 (1.5)	1.7 ± 0.9	1-5	1.2	0.8
	**Q4**	Drinking (Q4)	1 (0.8)	2.4 ± 1.4	1-5	0.6	-1.0
	**Q5**	Afraid of choking (Q5)	1 (0.8)	1.5 ± 0.9	1-5	1.8	2.4
	**Q6**	Hard to eat due to choking (Q6)	4 (3.1)	1.2 ± 0.5	1-4	3.2	10.6
	**Q7**	Pace of eating (Q7)	-	3.8 ± 1.5	1-5	-0.8	-0.8
	**Q8**	Vomiting (Q8)	28 (21.5)	1.4 ± 1.0	1-5	2.8	6.7
	**Q9**	Loneliness (Q9)	1 (0.8)	2.0 ± 1.3	1-5	1.0	-0.4
	**Q10**	Explaining to others (Q10)	1 (0.8	1.7 ± 1.1	1-5	1.2	0.2
	**Q11**	Name-calling (Q11)	2 (1.5)	1.2 ± 0.6	1-4	3.3	10.9
	**Q12**	Staring (Q12)	1 (0.8)	1.6 ± 1.0	1-5	1.7	2.6
	**Q13**	Scars (Q13)	4 (3.1)	1.5 ± 1.0	1-5	1.9	3.1
	**Q14**	Saying mean things (Q14)	-	1.2 ± 0.5	1-4	3.0	10.1
	**Q15**	Feeling awkward (Q16)	1 (0.8)	1.6 ± 0.9	1-5	1.3	0.7
	**Q16**	Feeling different (Q17)	2 (1.5)	1.4 ± 0.8	1-5	2.5	6.8
	**Q17**	Adjusting cloths (Q18)	1 (0.8)	1.3 ± 0.8	1-5	2.9	7.7
	**Q18**	Visible scars (Q19)	3 (2.3)	1.4 ± 0.9	1-5	2.5	5.6
	**Q19**	Feeling imperfect (Q20)	1 (0.8)	1.2 ± 0.6	1-5	3.8	17.4
	**Q20**	Smaller than peers (Q21)	28 (21.5)	1.4 ± 1.0	1-5	2.2	4.0
	**Q21**	Breathing difficulties (Q23)	3 (2.3)	2.0 ± 1.2	1-5	0.8	-0.3
	**Q22**	Sleeping (Q24)	2 (1.5)	1.5 ± 0.8	1-4	1.2	0.0
	**Q23**	Worried about future (Q25)	4 (3.1)	1.2 ± 0.5	1-4	3.1	11.5
	**Q24**	Sad (Q26	3 (2.3)	1.2 ± 0.5	1-3	2.5	5.7

Supplementary Table S5. Feasibility of the EA-QOL© questionnaire for 8-17 year old children (proxy-report, n=136 and self-report, n=130). The bold item numbers correspond with the items of the Dutch EA-QOL questionnaire. The item numbers number in brackets after the topics correspond with the items of the original Swedish-German pilot questionnaire before item reduction. The complete English questions can be found in the supplementary material of the original article[5]. Items were answered on a 5-point Likert scale, ranging from never (1) to always (5). Raw, untransformed scores are presented in this table. Feasibility (percentage of items with >5% missing values[5]) was considered poor (>30%), moderate (10-30%) or good (<10%).

File S5. Reliability of the field test

	Child-parent pairs (n)	Level of agreement, ICC (95% CI)
Eating	122	0.78 (0.71-0.84)
Social relationships	126	0.69 (0.59-0.77)
Body perception	125	0.76 (0.67-0.83)
Health and well-being	124	0.67 (0.57-0.75)
Total score	128	0.81 (0.74-0.86)

Supplementary Table S6. Comparison reliability (child-parent agreements) between proxy-reports and self-reports of the EA-QOL© questionnaire for 8-17 years old. The level of agreement can be considered poor (<0.50), moderate (0.50-0.74), good (0.75-0.90), or excellent (>0.90)[6]. ICC = intra-class correlation coefficient, CI = confidence interval.

File S6. Results of the PedsQL questionnaire

	2-7 year proxy-reports (n=100)	8-17 year olds proxy-reports (n=135)	8-17 year olds self-reports (n=130)
	Median (IQR)	*Median (IQR)*	*Median (IQR)*
Physical functioning	93.75 (81.25-100.00)	93.75 (84.38-100.00)	93.75 (87.50-100.00)
Emotional functioning	75.00 (65.00-90.00)	90.00 (75.00-100.00)	90.00 (80.00-100.00)
Social functioning	95.00 (75.00-100.00)	100.00 (85.00-100.00)	100.00 (90.00-100.00)
School functioning	90.00 (71.25-100.00)	80.00 (60.00-100.00)	80.00 (70.00-95.00)
Psychosocial Health	84.17 (71.67-95.00)	85.00 (76.67-96.67)	88.33 (78.33-96.67)
Total score	85.87 (76.11-94.14)	88.04 (80.43-96.74)	89.13 (82.61-96.74)

Supplementary Table S7. Subscale and total scores of the previously validated PedsQL questionnaire.

References

1. Dellenmark-Blom M, Dingemann J, Witt S, et al: The Esophageal-Atresia-Quality-of-life Questionnaires: Feasibility, Validity and Reliability in Sweden and Germany. J Pediatr Gastroenterol Nutr 67:469-477, 2018
2. Varni JW, Seid M, Kurtin PS: PedsQL 4.0: reliability and validity of the Pediatric Quality of Life Inventory version 4.0 generic core scales in healthy and patient populations. Med Care 39:800-812, 2001
3. Wild D, Grove A, Martin M, et al: Principles of Good Practice for the Translation and Cultural Adaptation Process for Patient-Reported Outcomes (PRO) Measures: report of the ISPOR Task Force for Translation and Cultural Adaptation. Value Health 8:94-104, 2005
4. DeMuro CJ, Lewis SA, DiBenedetti DB, et al: Successful implementation of cognitive interviews in special populations. Expert Rev Pharmacoecon Outcomes Res 12:181-187, 2012
5. Dellenmark-Blom M, Abrahamsson K, Quitmann JH, et al: Development and pilot-testing of a condition-specific instrument to assess the quality-of-life in children and adolescents born with esophageal atresia. Dis Esophagus 30:1-9, 2017
6. Koo TK, Li MY: A Guideline of Selecting and Reporting Intraclass Correlation Coefficients for Reliability Research. J Chiropr Med 15:155-163, 2016
